# Stability-Indicating Liquid Chromatographic Method for the Quantification of the New Antipsychotic Agent Asenapine in Bulk and in Pharmaceutical Formulation

**DOI:** 10.3797/scipharm.1112-07

**Published:** 2012-04-01

**Authors:** Usmangani K. Chhalotiya, Kashyap K. Bhatt, Dimal A. Shah, Jigar R. Patel

**Affiliations:** 1 Indukaka Ipcowala College of Pharmacy, Beyond GIDC Phase V, Vithal Udyognagar, New Vallabh Vidyanagar-388121, Anand, Gujarat, India; 2 Sun Pharmaceutical Industries Ltd, Baroda, Gujarat, India

**Keywords:** Asenapine, Reversed phase liquid chromatography, RP-HPLC, Forced degradation, Validation, Saphris, Sycrest

## Abstract

A simple, specific and stability-indicating reversed phase high performance liquid chromatographic method was developed for the quantitative determination of asenapine in tablet dosage form. A SunFire C_18_, 5 μm column having 250×4.6 mm i.d. in isocratic mode, with mobile phase containing 0.02 M potassium dihydrogen phosphate: acetonitrile (95:05, v/v, pH 3.5 adjusted with 1% *o*-phosphoric acid) was used. The flow rate was 1.0 mL min^−1^ and effluents were monitored at 232 nm. The retention time of asenapine was 5.51 min. The linearity for asenapine was in the range of 0.1–20 μg/ml. The recoveries obtained for asenapine were 98.31–101.51%. Asenapine stock solutions were subjected to acid and alkali hydrolysis, chemical oxidation, sunlight and dry heat degradation. The degraded product peaks were well resolved from the pure drug peak with significant difference in their retention time values. Stressed samples were assayed using developed LC method. The proposed method was validated with respect to linearity, accuracy, precision and robustness. The method was successfully applied to the estimation of asenapine in tablet dosage form.

## Introduction

**Fig. 1.**
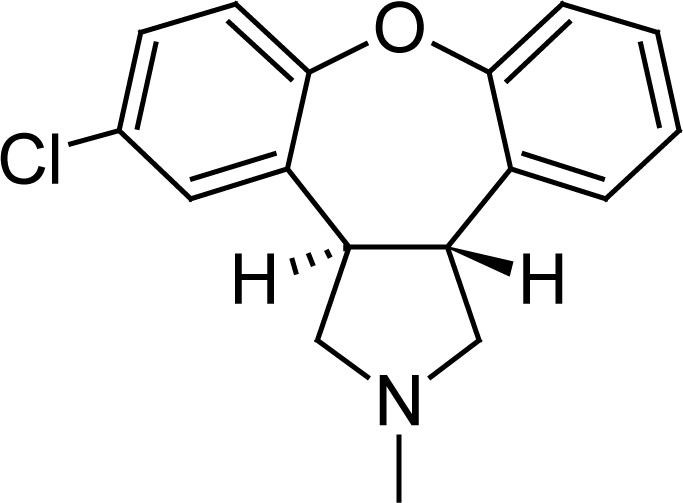
Chemical structure of Asenapine

Asenapine maleate (Org 5222, ASP) is a novel dibenzoxepinopyrrole [*rel*-(3a*R*,12b*R*)-5-chloro-2-methyl-2,3,3a,12b-tetrahydro-1*H*-dibenzo[2,3:6,7]oxepino[4,5-*c*]pyrrole (2*Z*)-but-2-enedioate] (Figure 1) with unique receptor pharmacology and is available as a fast-dissolving tablet for sublingual administration. It has potent dopaminergic (D_1_–D_4_), serotonergic (5-HT_2A_, 5-HT_2C_, 5-HT_6_ and 5-HT_7_), adrenergic (α_1_ and α_2_) and histaminergic (H_1_) activity, but it lacks significant anti-muscarinic activity [1]. ASP is an atypical antipsychotic approved in the USA in adults for the treatment of schizophrenia and for the acute treatment, as monotherapy or adjunctive therapy to lithium or valproate, of manic or mixed episodes associated with bipolar I disorder [2]. In the European Union ASP is associated with the treatment of moderate to severe manic episodes associated with bipolar I disorder in adults [3] (European Medicines Agency, 2010). In short-term trials, ASP has demonstrated superiority over placebo in the treatment of schizophrenia [4, 5] and acute manic episodes associated with bipolar I disorder [6, 8]. The proposed metabolism of ASP and the excretion profiles were recently published [9].

Literature survey revealed that asenapine and three metabolites were estimated in human plasma by LC/MS method [10]. Literature survey revealed that no method has been reported for the estimation of ASP in pharmaceutical dosage form. Forced degradation studies are an important way to know the possible route of degradation of pharmaceutical drugs. LC is the preferred method for the analysis of stability samples compared to UV and HPTLC method. As per ICH guidelines, a stability-indicating method should be applicable in stress conditions like acid base hydrolysis, oxidative stress study, dry heat degradation and photo degradation study. In the present study an attempt has been made to develop a stability-indicating liquid chromatographic method for the quantification of ASE in pharmaceutical dosage form [11–15].

## Results and Discussion

### Optimization of mobile phase

The objective of the method development was to resolve chromatographic peaks for active drug ingredients and degradation products produced under stress conditions with less asymmetric factor.

Various mixtures containing aqueous buffer, methanol and acetonitrile were tried as mobile phases in the initial stage of method development. Mixture of methanol: water (90:10, v/v), methanol-water (60:40, v/v), methanol: water (20: 80), acetonitrile-water (50:50, v/v), 0.02 M KH2PO4: methanol (70:30, v/v), 0.02 M KH2PO4: methanol (90:10, v/v, pH adjusted to 7.5 with 1 % TEA), were tried as mobile phase but satisfactory resolution of drug and degradation peaks were not achieved.

**Fig.2.**
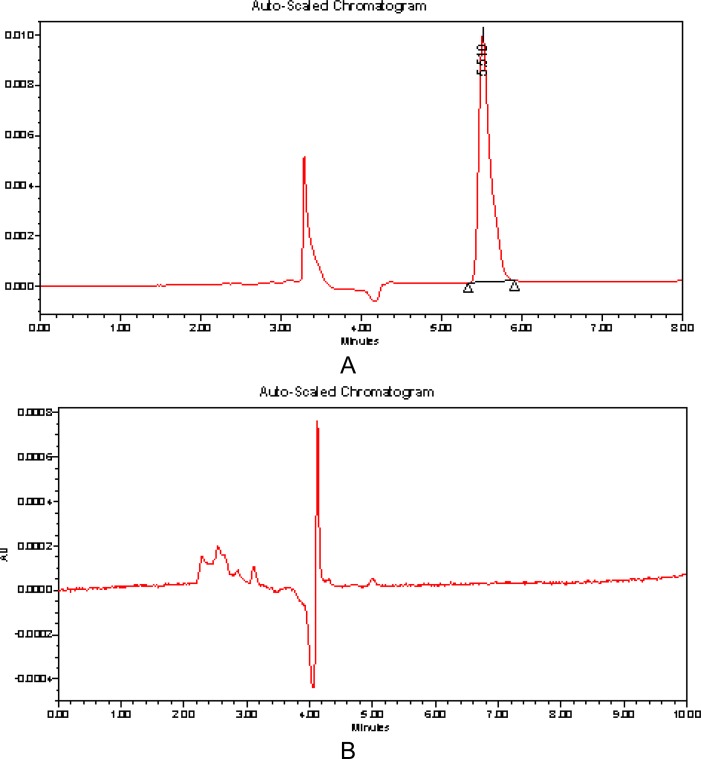
(A) Liquid chromatogram of ASP (RT 5.510) (B) Liquid chromatogram of placebo on C_18_ SunFire column using 0.01 M potassium dihydrogen phosphate buffer: Acetonitrile (pH 3.5 adjusted with orthophosphoric acid) (95:05, v/v) as the mobile phase

The mobile phase 0.02M potassium dihydrogen phosphate: acetonitrile (95:05, v/v, pH 3.5 adjusted with O - phosphoric acid) was found to be satisfactory and gave symmetric peak for ASP. The retention time for proposed method was found to be 5.5 min as shown in Figure. 2. The system suitability parameters like theoretical plates per meter and asymmetric factor for ASP were found to be 3805vand 0.67, respectively. The mobile phase flow rate was maintained at 1 mL min^−1^. The UV spectra of the drug showed that ASP absorbed appreciably at 232 nm, so detection was carried out at 232 nm.

### Validation of the Proposed Methods

#### Linearity

The calibration curve for ASP was found to be linear in the range of 0.1–20 μg mL^−1^ with a correlation coefficient of 0.9998. The standard deviation value of slope and intercept of ASP was found to be 842.13 and 361.27, respectively, which indicated strong correlation between peak area and concentration. The regression equation of calibration curves was obtained as y = 9112.5x + 45.098.

#### Precision

Instrument precision was determined by performing injection repeatability test and the RSD value for ASP was found to be 0.77%. The intra-day and inter-day precision studies were carried out and the RSD value was found to be 0.76–1.0% and 0.93–1.32%, respectively. The low RSD values indicate that the method is precise.

#### Accuracy

The accuracy of the method was determined by calculating recoveries of ASP by method of standard addition. The recoveries found to be 98.31–101.51% for ASP (table 1). The high values indicate that the method is accurate.

#### Limit of detection and limit of quantification

The detection limit and quantitation limit for ASP was 0.05 μg mL^−1^ and 0.1 μg mL^−1^, respectively. The above data shows that a nanogram quantity of the drug can be accurately and precisely determined.

#### Specificity

The specificity study was carried out to check the interference from the excipients used in the formulation by preparing synthetic mixture containing the drug and excipients. The chromatogram showed peaks for the drug without any interfering peak.

#### Forced degradation study

**Fig. 3.**
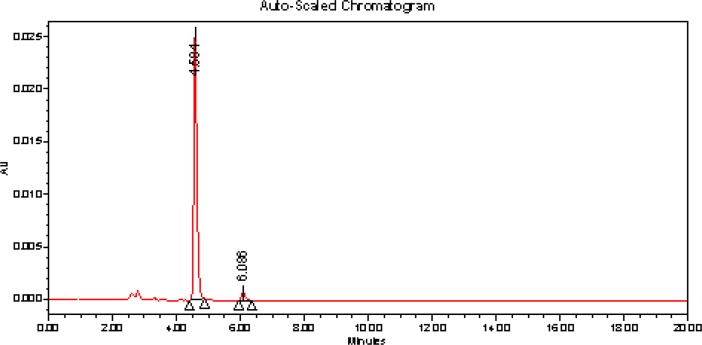
Chromatogram of 1M NaOH treated ASP at 80°C temperature for 24 h reflux.

Chromatogram of base hydrolysis performed at 80°C for 24 h reflux showed degradation of ASP with degradation product peak at retention time (RT) 6.086 min (Figure 3).

**Fig. 4.**
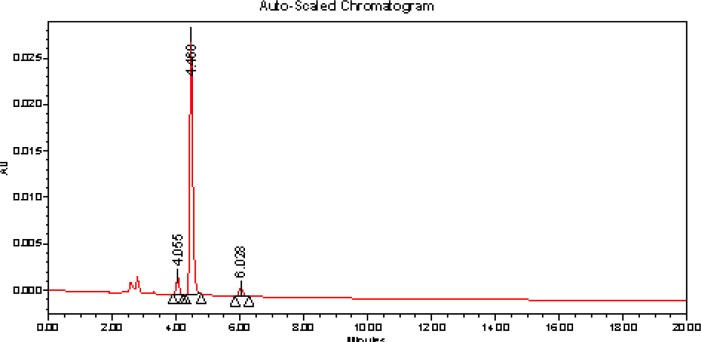
Chromatogram of 1M HCl treated ASP at 80°C temperature for 24 h reflux.

The chromatogram of acid hydrolysis performed at 80°C for 24 h reflux showed degradation of ASP with degradation product peak at retention time (RT) 4.055 min and 6.028 (Figure 4). The chromatogram of oxidized ASP with 3% hydrogen peroxide at 80°C for 24 h reflux showed drug was found to be stable and the chromatogram of ASP with dry heat at 80°C for 24 h showed drug was found to be stable. The chromatogram of ASP expose to sunlight for 24 h showed drug was found to be stable

The degradation study thereby indicated that ASP was found to be stable to oxidation (3% hydrogen peroxide), dry heat degradation study, effect of sunlight while it was susceptible to base hydrolysis and acid hydrolysis (table 2). No degradation products from different stress conditions affected determination of ASP.

#### Solution stability

The solution stability study showed that ASP was evaluated at room temperature for 24 h. The relative standard deviation was found to be below 2.0%. It showed that solutions were stable up to 24 h at room temperature.

#### Robustness

The method was found to be robust, as small but deliberate changes in the method parameters have no detrimental effect on the method performance as shown in Table 3. The low value of relative standard deviation indicated that the method was robust.

### Analysis of marketed formulations

The proposed method was successfully applied to the determination of ASP in their tablet dosage form (Tablet A). The % recovery for ASP was found to be 99.18 ± 0.56 mean value ± standard deviation of three determinations which was comparable with the corresponding labeled amounts.

## Conclusion

The proposed study describes stability-indicating LC method for the estimation of ASP in bulk and their pharmaceutical dosage form. The method was validated and found to be simple, sensitive, accurate and precise. Statistical analysis proved that method was repeatable and selective for the analysis of ASP without any interference from the excipients. The method was successfully used for determination of drug in their pharmaceutical formulation. Also, the above results indicate the suitability of the method for acid, base, oxidation, dry heat and photolytic degradation study. As the method separates the drugs from its degradation products, it can be used for analysis of stability samples. The method is suitable for the routine analysis of ASP in tablets. In addition, the HPLC procedure can be applied to the analysis of samples obtained during accelerated stability experiments to predict expiration dates of pharmaceuticals.

## Experimental

### Apparatus

The liquid chromatographic system of waters (Calcutta, India) containing 515 HPLC isocratic pump, variable wavelength programmable 2998 photodiode array detector and rheodyne injector with 20 μl fixed loop was used. A SunFire C_18_ column with 250×4.6 mm i.d. and 5 μm particle size was used as stationary phase.

### Reagents and materials

Analytically pure ASP was procured as gratis sample from Sun Pharmaceutical Pvt. Ltd., (Baroda, India). Methanol, water (E. Merck, Mumbai, India) was of LC grade, while potassium dihydrogen phosphate and ortho-phosphoric acid (S.D. fine chemicals, Mumbai, India) were of analytical grade and used for the preparation of mobile phase. Tablet formulation A (Asenapt (5mg), Sun pharmaceuticals Ltd., Sikkim, India) containing labeled amount of 5 mg of asenapine sublingual tablets was purchased from local market.

### Preparation of mobile phase and stock solution

Mobile phase was prepared by accurately weighing 0.340 g of potassium dihydrogen phosphate and dissolving in 100 ml of water. 95ml of buffer was mixed with 5 ml of acetonitrile, and pH was adjusted to pH 3.5 using 1 % *o*-phosphoric acid (OPA). The solution was filtered with Whatman filter paper No. 42 (0.45 μm). The solution was sonicated for 15 min for degassing prior to use.

Stock solutions were prepared by accurately weighing 10 mg of ASP and transferring to 10 ml volumetric flasks containing 3 ml of methanol. The flasks were sonicated for 10 min to dissolve the solids. Volumes were made up to the mark with methanol, which gave 1000 μg/ml. Aliquots from the stock solutions were appropriately diluted with mobile phase to obtain working standards of 100 μg/ml of drug.

### Chromatographic conditions

A reversed phase C_18_ column (SunFire) equilibrated with mobile phase comprising of 0.025M potassium dihydrogen phosphate: acetonitrile (95: 05, pH 3.5) was used. Mobile phase flow rate was maintained at 1 ml/ min and effluent was monitored at 232 nm. A 20 μL of sample was injected using a fixed loop, and the total run time was 8 min. All the chromatographic separations were carried out at controlled room temperature (25 ± 2 °C).

### Calibration curves for ASP

Appropriate aliquots of ASP working standard solution were taken in different 10 ml volumetric flasks. The volume was made up to the mark with mobile phase to obtain final concentrations of 0.1, 0.5, 1, 5, 10 and 20 μg/ml of ASP, respectively. The solutions were injected using a 20 μL fixed loop system and chromatograms were recorded. Calibration curves were constructed by plotting peak area versus concentrations of the drug and regression equations were computed for ASP.

### Analysis of Marketed Formulations

Twenty tablets were weighed accurately and finely powdered. Tablet powder equivalent to 5 mg ASP was put in 10 ml volumetric flask. A few ml of methanol were added to the above flask and the flask was sonicated for 15 min. The solution was filtered in another 10 ml volumetric flask using Whatman filter paper No. 42 and volume was made up to the mark with the same solvent.

Appropriate volume of the aliquot was transferred to a 10 ml volumetric flask and the volume was made up to the mark with the mobile phase to obtain a solution containing 5 μg/ml of ASP. The solution was sonicated for 10 min. It was injected as per the above chromatographic conditions and peak area was recorded. The quantifications were carried out by keeping these values to the linear equation of calibration curve.

### Validation

The method was validated for accuracy, precision, specificity, detection limit, quantitation limit and robustness.

### Accuracy

The accuracy of the method was determined by calculating recoveries of ASP by method of standard additions. Known amount of ASP (0, 2.5, 5, 7.5 μg/ml) was added to a pre quantified sample solutions and the amount of ASP was estimated by measuring the peak area and by fitting these values to the straight-line equation of calibration curve.

### Precision

The instrument precision was evaluated by injecting the solution containing ASP (5 μg/ml) three times repeatedly and peak area was measured. The results are reported in terms of % relative standard deviation. The intra-day and inter-day precision study of ASP was carried out by estimating the corresponding responses 3 times on the same day and on 3 different days (first, second and third day) for 3 different concentrations of ASP (0.1, 5, 20 μg/ml) and the results are reported in terms of % relative standard deviation (RSD).

### Specificity

The specificity was estimated by spiking commonly used excipients (starch, talc and magnesium stearate) into a pre weighed quantity of drug. The chromatogram was taken by appropriate dilutions and the quantities of drugs were determined.

### Limit of detection and quantification

The detection limit is defined as the lowest concentration of an analyte that can reliably be differentiated from background levels. Limit of quantification of an individual analytical procedure is the lowest amount of analyte that can be quantitatively determined with suitable precision and accuracy. LOD and LOQ were calculated using the following equation as per ICH guidelines. LOD = 3.3 × σ /S and LOQ = 10 × σ /S, where σ is the standard deviation of y-intercepts of regression lines and S is the slope of the calibration curve.

### Robustness

Robustness of the method was studied by deliberately changing the experimental conditions like flow rate, percentage of organic phase, pH of mobile phase and also by observing the stability of the sample solution at 25 ± 2° for 24 h. The sample solution was assayed at every 2 h interval up to 24 h.

### Forced degradation study

Stress degradation study using acid and alkali hydrolysis, chemical oxidation, exposure to sunlight and dry heat degradation was carried out and interference of the degradation products was investigated. ASP was weighed (10 mg) and transferred to 10 ml volumetric flasks and exposed to different stress conditions.

#### Alkali hydrolysis

To the 10 ml volumetric flask, 10 mg of ASP was added and 2 ml of 1 N NaOH was added to perform base hydrolysis. The flask was heated at 80°C for 24 h and allowed to cool to room temperature. Solution was neutralized with 1 N HCl and volume was made up to the mark with methanol. Appropriate aliquots were taken from the above solution and diluted with mobile phase to obtain final concentration of 20μg mL^−1^ of ASP.

#### Acid hydrolysis

To the 10 ml volumetric flask, 10 mg of ASP was added and 2 ml of 1 N HCl was added to perform acid hydrolysis. The flask was heated at 80°C for 24 h and allowed to cool to room temperature. Solution was neutralized with 1 N NaOH and volume was made up to the mark with methanol. Appropriate aliquot was taken from the above solution and diluted with mobile phase to obtain final concentration of 20μg mL^−1^ of ASP.

#### Oxidative stress degradation

To perform oxidative stress degradation, 10mg of ASP was added to 10 ml volumetric flask and 2 ml of 3% hydrogen peroxide was added. The mixture was heated in a water bath at 80°C for 2 h. and allowed to cool to room temperature and volume was made up to the mark with methanol. Appropriate aliquot was taken from above solution and diluted with mobile phase to obtain final concentration of 20 μg mL^−1^ of ASP.

#### Dry heat degradation

Analytically pure 10 mg sample of ASP was exposed in oven at 80°C for 24 h. The solids were allowed to cool and transferred to volumetric flasks (10 ml) and dissolved in a few ml of methanol. Volume was made up to the mark with the methanol. Solution was further diluted by mobile phase taking appropriate aliquots in 10 ml volumetric flask to obtain final concentration of 20 μg mL^−1^ of ASP.

#### Photolytic degradation

To study photolytic (sunlight) degradation, 10 mg of drug were exposed to sunlight for 24 h. The solids were allowed to cool and transferred to volumetric flask (10 ml) and dissolved in a few ml of methanol. Volume was made up to the mark with the methanol. Solution was further diluted with the mobile phase taking appropriate aliquots in 10 ml volumetric flask to obtain final concentration of 20 μg mL^−1^ of ASP.

All the reaction solutions were injected in the liquid chromatographic system and chromatograms were recorded.

## Figures and Tables

**Tab. 1.** Accuracy study for asenapine proposed LC method

**Amount of Sample (μg/ml)**	**Sets**	**Amount drug of spiked (μg/ml)**	**Area (n=3)**	**Average amount recovered (μg/ml)**	**% Recovery**	**Mean % Recovery**	**% RSD**
5	1	0	44514	4.92	97.60	98.31	0.81
	2	0	45230		99.17		
	3	0	44773		98.17		

5	1	2.5	69437	7.53	102.30	100.53	1.27
	2	2.5	68751		100.80		
	3	2.5	67709		98.51		

5	1	5	91313	9.998	100.31	99.97	1.04
	2	5	90142		97.74		
	3	5	92014		101.85		

5	1	7.5	114004	12.75	100.12	101.51	1.23
	2	7.5	113664		99.37		
	3	7.5	116252		105.05		

**Tab. 2.** Data derived from forced degradation study for asenapine proposed LC method

**Condition**	**Time (h)**	**% Recovery ASP**	**Retention time of degradation products**
Base 1 N NaOH	24	91.70	6.086
Acid 1 N HCl	24	82.41	4.055, 6.028
3% hydrogen peroxide	24	98.46	–
Dry heat*	24	99.31	–
Sunlight	24	99.07	–

* Samples were heated at 80°C for specified period of time.

**Tab. 3.** Data derived from robustness for asenapine proposed LC Method

**Parameters**	**Normal condition**	**Change in condition**	**Change in % RSD**
Flow Rate	1.0 ml/min	0.9 ml/min	0.97
		1.1 ml/min	1.06

pH	3.5	3.0	0.87
		4.0	1.30
Mobile phase ratio	95:05	93:07	0.92
		97:03	1.35

## References

[1] Shahid M, Walker GB, Zorn SH, Wong EH (2009). Asenapine: a novel psychopharmacologic agent with a unique human receptor signature. J Psychopharmacol.

[2] Saphris®. Asenapine sublingual tablets Full prescribing information Schering Corporation, a subsidiary of Merck & Co. Inc. 2010. Whitehouse Station, NJ0. http://www.spfiles.com/pisaphrisv1.pdf [accessed January 2011].

[3] European Medicines Agency http://www.ema.europa.eu/ema/index.jsp?curl=pages/medicines/human/medicines/001177/human_med_001379.jsp&murl=menus/medicines/medicines.jsp&mid=WC0b01ac058001d124&jsenabled=true.

[4] Potkin SG, Cohen M, Panagides J (2007). Efficacy and tolerability of asenapine in acute schizophrenia: a placebo- and risperidone-controlled trial. J Clin Psychiatry.

[5] Kane JM, Cohen M, Zhao J, Alphs L, Panagides J (2010). Efficacy and safety of asenapine in a placebo- and haloperidol-controlled trial in patients with acute exacerbation of schizophrenia. J Clin Psychopharmacol.

[6] McIntyre RS, Cohen M, Zhao J, Alphs L, Macek TA, Panagides J (2009). A 3-week, randomized, placebo-controlled trial of asenapine in the treatment of acute mania in bipolar mania and mixed states. Bipolar Disord.

[7] McIntyre RS, Cohen M, Zhao J, Alphs L, Macek TA, Panagides J (2009). Asenapine versus olanzapine in acute mania: a double-blind extension study. Bipolar Disord.

[8] McIntyre RS, Cohen M, Zhao J, Alphs L, Macek TA, Panagides J (2010). Asenapine in the treatment of acute mania in bipolar I disorder: a randomized, double-blind, placebo-controlled trial. J Affect Disord.

[9] van de Wetering-Krebbers SF, Jacobs PL, Kemperman GJ, Spaans E, Peeters PA, Delbressine LP, van Iersel ML (2011). Metabolism and excretion of asenapine in healthy male subjects. Drug Metab Dispos.

[10] de Boer T, Meulman E, Meijering H, Wieling J, Dogterom P, Lass H (2012). Quantification of asenapine and three metabolites in human plasma using liquid chromatography–tandem mass spectrometry with automated solid-phase extraction: application to a phase I clinical trial with asenapine in healthy male subjects. Biomed Chromatogr.

[11] Topic Q1B Photo stability Testing of New Drug Substances and Products. International Conference on Harmonization (ICH), IFPMA, Geneva, 1996

[12] Topic Q2 (R1) Validation of Analytical Procedure; Test and Methodology. International Conference on Harmonization (ICH), Geneva, 2005

[13] Topic Q2A Validation of Analytical Procedures: Consensus Guidelines. International Conference on Harmonization (ICH), Geneva, 1994

[14] Topic Q2B Validation of Analytical Procedures: Methodology, Consensus Guidelines. International Conference on Harmonization (ICH), Geneva, 1996

[15] Topic Q1A (R2) Stability Testing of New Drug Substances and Products. International Conference on Harmonization (ICH), IFPMA, Geneva, 2003

